# A population-based study of the appearances of enteric *Campylobacter* and non-typhoidal *Salmonella* infections on computed tomography

**DOI:** 10.1080/07853890.2024.2356638

**Published:** 2024-05-22

**Authors:** Oskar Ljungquist, Sophie Poijes, Torgny Sunnerhagen, Anna Bläckberg

**Affiliations:** a Department of Clinical Sciences, Division of Infection Medicine, Lund University, Lund, Sweden; b Department of Infectious Diseases, Helsingborg Hospital, Helsingborg, Sweden; c Department of Radiology, Helsingborg Hospital, Helsingborg, Sweden; d Clinical Microbiology, Infection Prevention and Control, Office for Medical Services, Lund, Sweden; e Department of Infectious Diseases, Skåne University Hospital, Lund, Sweden

**Keywords:** Diagnostic medicine, foodborne infections, diarrhea, colitis, radiology

## Abstract

**Background:**

Swift identification and diagnosis of gastrointestinal infections are crucial for prompt treatment, prevention of complications, and reduction of the risk of hospital transmission. The radiological appearance on computed tomography could potentially provide important clues to the etiology of gastrointestinal infections. We aimed to describe features based on computed tomography of patients diagnosed with *Campylobacter, Salmonella* or *Shigella* infections in South Sweden.

**Methods:**

This was a retrospective observational population-based cohort study conducted between 2019 and 2022 in Skåne, southern Sweden, a region populated by 1.4 million people. Using data from the Department of Clinical Microbiology combined with data from the Department of Radiology, we identified all patients who underwent computed tomography of the abdomen CTA two days before and up to seven days after sampling due to the suspicion of *Campylobacter, Salmonella* or *Shigella* during the study period.

**Results:**

A total of 215 CTAs scans performed on 213 patients during the study period were included in the study. The median age of included patients was 45 years (range 11–86 years), and 54% (114/213) of the patients were women. Of the 215 CTAs, 80% (*n* = 172) had been performed due to *Campylobacter* and 20% (*n* = 43) due to *Salmonella* enteritis. CTA was not performed for any individual diagnosed with *Shigella* during the study period. There were no statistically significant differences in the radiological presentation of *Campylobacter* and *Salmonella* infections.

**Conclusion:**

The most common location of *Campylobacter* and *Salmonella* infections was the cecum, followed by the ascending colon. Enteric wall edema, contrast loading of the affected mucosa, and enteric fat stranding are typical features of both infections. The CTA characteristics of *Campylobacter* and *Salmonella* are similar, and cannot be used to reliably differentiate between different infectious etiologies.

## Introduction

The global burden of gastrointestinal infections is significant, with an estimated 1.7 billion cases of childhood diarrheal disease every year according to the World Health Organization (WHO) [1]. Each year, foodborne illnesses caused by *Campylobacter,* non-typhoidal *Salmonella* and *Shigella* are estimated to cause 96, 78, and 51 million episodes, respectively [2]. These infections are linked to contaminated water and food sources, insufficient hygiene practices, and poor sanitation and are more prevalent in developing countries. *Campylobacter,* non-typhoidal *Salmonella* and *Shigella* have a wide spectrum of clinical presentations ranging from mild self-limiting gastroenteritis to severe invasive disease and death. In 2019, non-typhoidal *Salmonella* caused approximately 215,000 deaths globally [3]. For patients with severe disease requiring hospitalization, computed tomography is sometimes performed during the work-up, as the clinical presentation of bacterial gastroenteritis can mimic other conditions, such as inflammatory bowel disease, drug-induced colitis, and ischemic colitis.

Previously, only a few reports on CT findings of gastrointestinal infections have been published, including only a few patients [4–6]. CT features have been suggested as indicative of specific bacteria [7]. Swift identification and diagnosis of gastrointestinal infections are crucial for prompt treatment, prevention of complications, and reduction in the risk of hospital transmission. In situations where microbiological results are prolonged or absent, the radiological appearance on computed tomography could potentially provide important clues to the etiology of gastrointestinal infection.

In this study, we aimed to describe features based on computed tomography of patients diagnosed with *Campylobacter, Salmonella* or *Shigella* infections in South Sweden. Additionally, we wanted to investigate if there were statistically significant differences in the radiological appearances between the different etiologies.

## Methods

### Study design and setting

This was a retrospective observational population-based cohort study conducted between 2019 and 2022 in Skåne, southern Sweden, a region populated by 1.4 million people. The Clinical Microbiology Laboratory in Lund is responsible for all the microbiological diagnostics in this region. Data were retrieved from stool cultures of all patients diagnosed with *Campylobacter, Salmonella* and *Shigella* in the region during the study period.

Data were retrieved from all patients who underwent computed tomography of the abdomen (CTA) with intravenous contrast during the study period from PACS IDS7 (Sectra, Linköping, Sweden). Using both registers, we identified all patients who underwent CTA two days before and up to seven days after being diagnosed with *Campylobacter, Salmonella* or *Shigella* during the study period. Patients could be included multiple times in the study if they presented multiple times with the same or different aetiology.

The intravenous contrast used in the study was Omnipaque 350 mg I/mL, dosed according to patients’ weight, with reduced dose in patients with impaired kidney function (estimated glomerular filtration rate <45 mL/min/1.73 m^2^) and/or older age (>70 years). Therefore, CTAs without intravenous contrast was not included. CTAs in which oral contrast was administered, in addition to intravenous contrast, were also included.

Patients who had previously undergone total colectomy were excluded, as were those with CTAs that could not be assessed due to severe motion artifacts. The medical records of all patients were reviewed using Melior software (Melior, Siemens Healthcare Service, Upplands Väsby, Sweden).

### Ethics

This study was approved by the Swedish Ethical Review Authority (DNR-2021-04866) as well as an institutional approval. This national authority waived the need for patient consent.

### Outcome

**Figure 1.**
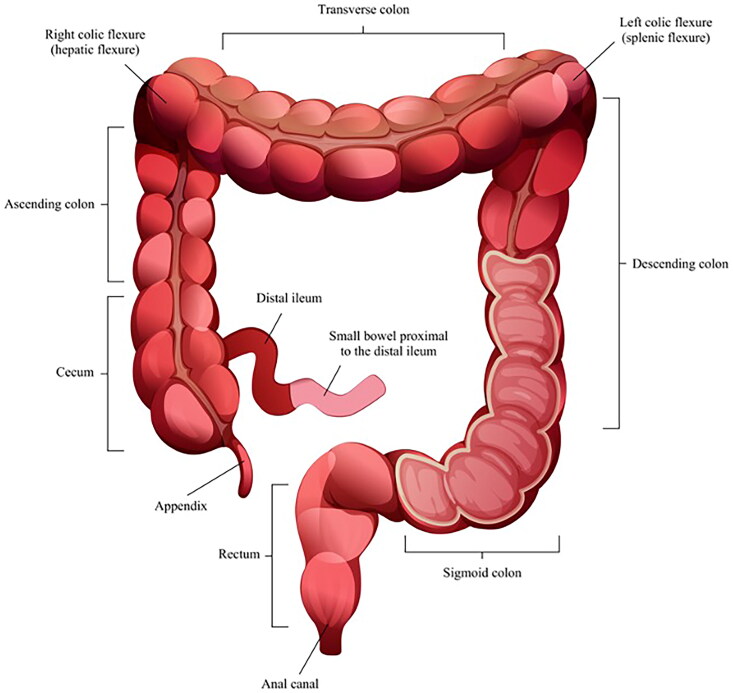
The different segments of the distal small bowel and the colon.

Each CTA was examined using a predefined study protocol with twenty different variables of interest. The distal small bowel and colon were divided into 11 different segments (Figure 1), and the presence of increased mesenteric vascular appearance (comb sign) and contrast loading of the affected mucosa and serosa were noted (yes or no). Additionally, the thickness of the most affected enteric wall was measured in millimeters, using the mean value of two random measurements. If edema was present or not (yes/no), any enlarged mesenteric lymph nodes were noted (no = <1 cm, yes ≥1 cm). Also, the presence or absence of ascites, enteric fat stranding and the presence or absence of skipped lesions was recorded. Pancolitis was defined as colitis affecting the cecum and rectum. The presence of a water halo sign and an empty colon sign, which has been previously described as a radiological appearance, was noted [8–10]. In addition, the presence of gas intramurally in the mesenterial vessels or abdomen was noted. Short-axis measurements and attenuation according to the Hounsfield unit were recorded for the largest lymph nodes.

The second author (SP) examined all CTAs blindly, with no information on the microbiological etiology of the included patients. In addition, C-reactive protein (CRP), peripheral total leukocyte counts, and temperature ± one day of CTA were extracted from the medical records.

### Microbiology

Gastrointestinal pathogens were detected in fecal samples using a culture-based method until February 2020 and with a PCR-directed culture method using Amplidiag (Mobidiag, Ltd.). To identify *Campylobacter* before the introduction of Amplidiag PCR, selective *Campylobacter*-agar (Neogen) was used, and CampyGen (Thermo Scientific) was used to achieve a microaerophilic environment. After the introduction of Amplidiag PCR, *Campylobacter* was only cultured from fecal samples upon specific requests. When the primary culture was used and when a PCR-directed culture was the main method, *Salmonella* and *Shigella were* cultured when detected. The culture was based on xylose-lysine-deoxycholate (XLD) agar, *Salmonella* CHROMAgar (CHROMAgar), and Rappaport broth used for enrichment of *Salmonella*. In addition to MALDI-TOF MS, agglutination using antisera (SSI Diagnostica for *Salmonella* and SIFIN Antisera for *Shigella*, earlier from Reagensia for both) was used for *Salmonella* and *Shigella* typing.

### Statistical analysis

Numerical data are presented as medians, means, and ranges, and qualitative data are presented as proportions (%). The chi-square test was used for categorical data, and the Student’s *t*-test and Mann–Whitney test were used for continuous variables. Pearson’s correlation test was used for correlation analysis. Statistical significance was set at *p* < 0.05. Statistical analyses were performed using Prism, version 7 (GraphPad Software).

## Results

### Patient characteristics

**Figure 2.**
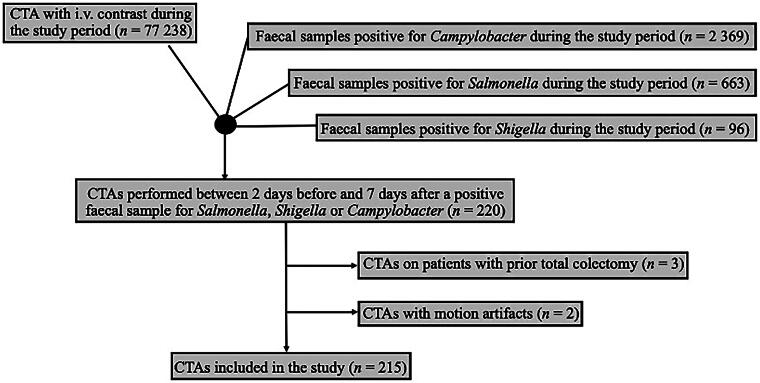
Flowchart of steps of inclusion.

In total, 215 CTAs performed on 213 patients with *Salmonella* and *Campylobacter* during the study period were included in the study (Figure 2). No patient with *Shigella* was included. The median age of included patients was 45 years (range 11–86) and 54% (114/213) of the patients were women. In total, 23 patients (11%) had previous gastric bypass surgery: 22 patients (13%) in the *Campylobacter* group, compared to one (2%) in the *Salmonella* group. In the *Campylobacter* group, five (3%) and one (1%) patients suffered from ulcerative colitis and Mb Crohn, respectively. In the *Salmonella* group, one patient (2%) was previously diagnosed with Mb Crohn (Table 1). Out Of the 215 CTAs, 80% (*n* = 172) had been performed due to *Campylobacter* and 20% (*n* = 43) to enteric *Salmonella* enteritis. Also, 19% of these (8/43) also had *Salmonella* growth in blood cultures, in addition to a *Salmonella*-positive fecal sample.

**Table 1.** Baseline characteristics.

Baseline characteristics	*N*
Age, years, (range)	45 (11–86)
Female sex (%)	114 (54)
Etiology	
*Campylobacter*	172 (80)
*Salmonella* spp.	43 (20)
Previous gastric bypass surgery (%)	23 (11)
Inflammatory bowel disease (%)	7 (4)
*Campylobacter*	6 (3)
*Salmonella* spp.	1 (2)
Previous partial colectomy (%)	5 (2)
*Campylobacter*	4 (2)
*Salmonella* spp.	1 (2)
Oral contrast	86 (40)

Out of the 172 CTAs performed due to *Campylobacter*, 76 (44%) were performed with oral contrast, in addition to intravenous contrast. For *Salmonella*, this number was 10 (23%).

None of the patients in whom CTA was performed due to *Campylobacter* had a bloodstream infection due to *Campylobacter*. Two CTAs were performed on one individual diagnosed with both *Campylobacter* and *Salmonella* during the study period, one year apart. One patient was subjected to two CTAs, five weeks apart due to *Campylobacter* infection.

CTA was not performed for any individual diagnosed with *Shigella* during the study period. No CTA was performed with intraperitoneal gas, ileus, or toxic megacolon during the study period. For both infections due to *Campylobacter* and *Salmonella*, several enteric segments were mostly involved (Table A1).

### Campylobacter and characteristics of CTA

In total, 170 (99%) of CTAs were pathological, according to a predefined study protocol. For enteric inflammation, the most common pathological location was the cecum (*n* = 151, 88%), followed by the ascending colon (*n* = 144, 84%), and the right colic flexure (*n* = 134, 78%) (Table 2). Increased mesenteric vascular appearance was seen in a minority of CTAs (*n* = 74, 43%), and whereas contrast loading of the affected mucosa was typical (*n* = 138, 80%), contrast loading of the affected serosa was less common (*n* = 116, 67%). Enteric wall edema was typical (*n* = 115, 67%) and the median thickness of the enteric wall was 4 mm (range 1.5–10 mm). Ascites and lymph nodes enlarged more than 1 cm were observed in less than half of the patients (*n* = 64, 38%; and *n* = 76, 44%, respectively).

**Table 2.** Enteric segment involved.

Segment involved	*Campylobacter*	*Salmonella*
Location	*n* (%)	*n* (%)
Small bowel proximal to the distal ileum	18 (10)	7 (16)
Distal ileum	128 (74)	29 (67)
Appendix	24 (14)	4 (9)
Cecum	151 (88)	32 (74)
Ascending colon	144 (84)	31 (72)
Right colic flexure	134 (78)	27 (63)
Transverse colon	117 (68)	26 (60)
Left colic flexure	97 (56)	23 (53)
Descending colon	103 (60)	22 (51)
Sigmoid colon	91 (53)	19 (44)
Rectum	85 (49)	19 (44)

Enteric fat stranding was frequent (*n* = 121, 70%), but the presence of skipped lesions (*n* = 16, 9%) were rare. For each patient, a median of seven enteric segments (range 0–11) was pathological in *Campylobacter* etiology.

### Salmonella and characteristics of CTA

In total, 42 (98%) of CTAs were pathological, according to the study protocol. For enteric inflammation, the most common pathological location was the cecum (*n* = 34, 74%), followed by the ascending colon (*n* = 31, 72%), and the distal ileum (*n* = 29, 67%) (Table 2). Increased mesenteric vascular appearance was frequent (*n* = 24, 56%), contrast loading of the affected mucosa was typical (*n* = 36, 84%), as well as contrast loading of the affected serosa (*n* = 29, 67%). The majority showed enteric wall edema (*n* = 29, 67%), and the median thickness of the enteric wall was 4 mm (range 1–8 mm). The presence of ascites and enlarged lymph nodes was observed in more than one third of the patients (*n* = 16, 37% and *n* = 15, 35%, respectively). Enteric fat stranding was commonly observed (*n* = 30, 70%), but skipped lesions were uncommon (*n* = 2, 4%). In total, a median of five enteric segments (range 0–10) were pathological in *Salmonella* CTAs. There was no statistical difference between the number of segments involved in CTAs in patients with *Salmonella* growth in blood cultures (median 7 segments), in addition to a *Salmonella*-positive fecal sample, compared to patients with *Salmonella*-positive fecal samples only (median 4 segments, *p* = 0.07).

### Comparison of Campylobacter and Salmonella aetiology

For the predefined study protocol variables, no statistically significant differences were observed in the radiological presentations of *Campylobacter* and *Salmonella* (Table 3).

**Table 3.** Comparison of CTA findings between *Campylobacter* and *Salmonella.*

	*Campylobacter n* (%)	*Salmonella n* (%)	*p-*Value
Increased mesenterial vascular appearance (comb sign)	74 (43)	24 (56%)	0.13
Contrast loading of the affected mucosa	138 (80)*	36 (84)^	0.6
Contrast loading of the affected serosa	116 (67)	29 (67)	1
Enteric wall oedema	115 (67)	29 (67)	0.9
Thickness of the enteric wall, mm (mean, range)	4 (1.5–10)¨	4 (1–8)’	0.9
Thickness of the enteric wall >3 mm	134 (78)	32 (74)	0.6
Intramural gas	0 (0)	0 (0)	–
Mesenterial vessels/v. porta gas	0 (0)	0 (0)	–
Free abdominal gas	0 (0)	0 (0)	–s
Enlarged mesenterial lymph nodes	76 (44)	15 (35)	0.3
Hounsfield unit value of largest lymph node, mm (range)	92 (30–148)	94 (45–139)	0.6
Short axis measurement largest lymph node, mm (range)	10 (5–18)	9 (4–15)	0.06
Ascites	64 (37)	16 (37)	0.9
Enteric fat stranding	121 (70)	30 (70)	0.9
Skipped lesions	16 (9)	2 (4)	0.3
No of segment involved, median (range)	7 (0–11)	5 (0–10)	0.1
Water halo sign	108 (63)	26 (60)	0.8
Empty colon sign	100 (58)	18 (42)	0.06
Pancolitis	72 (42)	13 (30)	0.2

*Nine examinations could not be assessed owing to oral contrast. ^Three examinations could not be performed. ¨Five examinations could not be assessed.’2 Examinations could not be performed. Pancolitis was defined as colitis affecting the cecum and rectum.

For *Campylobacter,* there was a statistically significant correlation between the number of segments involved and crp (*r* = 0.2, *p* = 0.009), but not with leukocyte count (*r* = 0.02, *p* = 0.8) or temperature (*r* = 0.1, *p* = 0.2) (Table 4). For *Salmonella,* there was no statistically significant correlation between the number of segments involved and crp (*r* = −0.1, *p* = 0.5), leukocyte count (*r* = −0.2, *p* = 0.3), or temperature (*r* = 0.1, *p* = 0.5).

**Table 4.** Further comparisons between *Campylobacter* and *Salmonella.*

	*Campylobacter n* (%)	*Salmonella n* (%)	*p-*Value
CRP (mg/L), median (range)	125.5 (10–333)	117 (0.6–478)	0.8
Leucocyte count × 10^9^/L	9.0 (3.7–28.3)	8.46 (3.0–21.6)	0.5
Temperature (degrees celsius)	37.6 (35.5–40.7)	37.8 (35.9–40.2)	0.2

CRP: C-reactive protein.

## Discussion

This population-based study in South Sweden aimed to describe features on computed tomography of patients diagnosed with enteric *Campylobacter, Salmonella* and *Shigella* infections in South Sweden and to investigate possible differences in the radiological appearances between the different etiologies. Since there were no patients with microbiological evidence of *Shigella*, only CTAs with symptoms of *Campylobacter* and *Salmonella* were included in the study. We found that 99% of the CTAs performed displayed pathological features, and the most common location of pathological features for both *Campylobacter* and *Salmonella* was the cecum, followed by the ascending colon. Contrast loading of the affected mucosa and serosa was commonly observed in both the infections. Enteric wall edema, enteric fat stranding, and water halo signs were typical for both infections. The radiological appearances of both *Campylobacter* and *Salmonella* infection*s.*

To our knowledge, our study is the most comprehensive account of the features of *Campylobacter* and *Salmonella* enteric infections on computed tomography, including a sizable cohort of patients compared to previous case reports and reviews. Previous reports have included between one hundred and seventeen cases [7–13].

Fat stranding on CT was common in both *Campylobacter* and *Salmonella* etiologies. Although fat stranding can occur in a variety of disorders within the abdomen, some studies indicate that fat stranding that is disproportionally more severe than the degree of wall thickening could suggest an inflammatory condition such as diverticulitis [14,15].

Our study is in agreement with previous studies (one case report, one review) locating *Campylobacter* and *Salmonella* enteric infections on the right side of the colon [4,13]. The rate of pancolitis was higher in our study (42% for *Campylobacter*, 30% for *Salmonella*) compared 26% in a previous study, but this study included colitis of any etiology [16].

Enlarged lymph nodes of both *Campylobacter* and *Salmonella* etiologies were observed in a minority of CTAs in our study, of both *Campylobacter* and *Salmonella* etiologies. Skipped lesions are uncommon manifestations of *Campylobacter* and *Salmonella* infections. Skip lesions are often observed in Crohn’s disease, a disease characterized by transmural inflammation that affects the entire gastrointestinal tract [17]. The radiological appearance of enteric infections can mimic the appearance of inflammatory bowel disease, and the clinical presentation can be similar. A ‘comb’ sign, together with small bowel involvement and enlarged lymph nodes could suggest inflammatory colitis, rather than infectious [8]. The same article found that an ‘empty colon’ sign, with a continuous distribution of inflammation and absence of enlarged lymph nodes, suggests infectious colitis. Our aim was not to compare radiological appearances between inflammatory bowel disease and *Campylobacter/Salmonella*, but our findings could be used to compare radiological characteristics of infectious colitis in future review articles.

In our study, ascites was a common manifestation of infection, which has been previously described in patients with blood cultures positive for *S. enterica* subsp*. enterica* ser. typhi, and *S. enterica* subsp*. enterica* ser. Paratyphi [6]: We did not include *Salmonella typhi* or *Salmonella paratyphi* in our study.

The characteristics of CTA of *Campylobacter* and *Salmonella* are similar and cannot reliably be used to differentiate between different infectious etiologies. We believe that fecal samples for the detection of gastrointestinal pathogens are essential to differentiate inflammatory bowel disease from gastrointestinal infections.

No patient underwent CTA due to *Shigella* enteric infection during the study period, we are therefore unable to report on CTA features of *Shigella*. To our knowledge, there are few previous studies addressing this.

The rate of CTAs performed in patients suffering from *Campylobacter* and *Salmonella* was 1:5, which is consistent with the numbers from the Public Health Agency of Sweden, which revealed that *Campylobacter* is five times more common than *Salmonella* [18].

For *Campylobacter*, we found a statistically significant correlation between C-reactive protein level and the spread of inflammation, but not with leukocyte count or temperature. It seems reasonable that enlarged inflammation is correlated with higher CRP; however, no such correlation was observed for CTAs performed due to *Salmonella*.

The strengths of our study include the population-based study design, covering three full years using two different registries for case finding, and the fact that the radiologist examined all CTAs blindly. The limitations of our study include the fact that CTAs were reviewed by only one radiologist. Ideally, two or more radiologists should have examined all CTAs blindly, with reported inter-rater reliability and agreement. A few variables were not explored when CTAs were examined, such as spleen enlargement. We could not analyze the serotypes of the pathogens to investigate any statistical associations between different serotypes and CT features, nor did we investigate patient demographics with respect to CT features.

A minority of the CTAs in our study were performed on patients with IBD (4%) and previous colectomy (2%), and 40% of CTAs were performed using oral contrast. If this is the case, our results are unlikely, but cannot be ruled out.

## Conclusion

The most common site of infection for both *Campylobacter* and *Salmonella* was the cecum, followed by the ascending colon. Enteric wall edema, contrast loading of the affected mucosa, and enteric fat stranding are typical features of both infections. The characteristics of CTA of *Campylobacter* and *Salmonella* are similar and cannot reliably be used to differentiate between different infectious etiologies.

## Data Availability

The participants of this study did not give written consent for their data to be shared publicly, so due to the sensitive nature of the research supporting data is not available.

## Appendix A.

**Table A1.** Number of enteric segments involved.

No of segments^#^ involved	*Campylobacter n* (%)	*Salmonella n* (%)
0	10 (6)	3 (7)
1	1 (1)	1 (2)
2	6 (3)	3 (7)
3	17 (10)	4 (5)
4	13 (8)	5 (12)
5	24 (14)	6 (14)
6	10 (6)	4 (9)
7	11 (9)	2 (5)
8	16 (9)	4 (5)
9	50 (29)	10 (23)
10	13 (8)	1 (2)
11	1 (1)	0 (0)

^#^Segments divided as denoted in Figure 1.
